# Senolytics: Eliminating Senescent Cells and Alleviating Intervertebral Disc Degeneration

**DOI:** 10.3389/fbioe.2022.823945

**Published:** 2022-03-02

**Authors:** Yuhao Wu, Shiwei Shen, Yifeng Shi, Naifeng Tian, Yifei Zhou, Xiaolei Zhang

**Affiliations:** ^1^ Department of Orthopaedics, The Second Affiliated Hospital and Yuying Children’s Hospital of Wenzhou Medical University, Wenzhou, China; ^2^ The Second School of Medicine, Wenzhou Medical University, Wenzhou, China; ^3^ Key Laboratory of Orthopaedics of Zhejiang Province, Wenzhou, China; ^4^ Chinese Orthopaedic Regenerative Medicine Society, Hangzhou, China

**Keywords:** senolytic, intervertebral disc degeneration, IVDD, senescence-associated secretory phenotype, SASP, cellular senescence, aging

## Abstract

Intervertebral disc degeneration (IVDD) is the main cause of cervical and lumbar spondylosis. Over the past few years, the relevance between cellular senescence and IVDD has been widely studied, and the senescence-associated secretory phenotype (SASP) produced by senescent cells is found to remodel extracellular matrix (ECM) metabolism and destruct homeostasis. Elimination of senescent cells by senolytics and suppression of SASP production by senomorphics/senostatics are effective strategies to alleviate degenerative diseases including IVDD. Here, we review the involvement of senescence in the process of IVDD; we also discuss the potential of senolytics on eliminating senescent disc cells and alleviating IVDD; finally, we provide a table listing senolytic drugs and small molecules, aiming to propose potential drugs for IVDD therapy in the future.

## Introduction

Intervertebral disc degeneration (IVDD) refers to an age-related change that mainly occurs in the lumbar intervertebral disc and often precedes other age-related changes (Adams and Roughley, 2006; Vergroesen et al., 2015). During the process of IVDD, annulus fibrosis (AF), one of the important compositions of intervertebral disc, loses its original layer and toughness, and reticulated degeneration and hyalinization appear, while the percentage of water decreases in another component called nucleus pulposus (NP). As a result, the intervertebral disc loses its normal elasticity and tension (Roberts et al., 2006). Severe trauma or repeated inconspicuous damage may cause the annulus fibrosis to become weak or even rupture. There are two results depending on the severity of damage to AF. If the annulus fibrosus is partially teared, the nucleus pulposus will protrude from the weak or ruptured area, compressing the nerve root and producing signs of nerve root damage; however, if the annulus fibrosus is completely ruptured, the broken nucleus pulposus tissue is likely to prolapse from the disc and enter into the spinal canal, which will cause irreversible damage to spinal cord (Roberts et al., 2006; Carrino et al., 2009).

IVDD is considered to be one of the main causes of low back pain and lumbar herniated disc (Brinjikji et al., 2015). As the life expectancy of the global population rises slowly, the incidence of IVDD will become increasingly high, thus resulting in a high economic burden (Smith et al., 2011). Although the situation is severe, the molecular mechanism of IVDD has not yet been fully clarified yet. At present, there are two main treatment strategies for IVDD: surgery and drug therapy. However, the main effects of existing drugs are inhibition of inflammation and post-onset analgesia, which cannot effectively prevent intervertebral disc from degenerating in the early stage (Wu et al., 2020).

Aging is the primary risk factor for the development of IVDD, which causes the accumulation of senescent cells in the intervertebral disc (Wang et al., 2016). Researchers have found that senescent NP cells play an important role in the initiation of IVDD (Zhang Y. et al., 2020). The number of senescent NP cells increased significantly during IVDD (Sakai et al., 2012; Dudek et al., 2017), suggesting the deleterious effect of senescent NP cells on the pathogenesis of IVDD. Recent studies have shown that senescent cells could secrete metabolic factors such as pro-inflammatory cytokines, matrix-degrading proteases, growth factors, and chemokines, which caused changes of the extracellular matrix (ECM) (Gruber et al., 2011; Zhao et al., 2011; Bedore et al., 2014). In addition, senescent cells can affect adjacent cells through paracrine, thereby inducing the catabolism and inflammation in the microenvironment of intervertebral disc. The metabolic factors secreted by senescent cells are collectively named senescence-associated secretory phenotype (SASP) (Ngo et al., 2017). In recent years, investigations on new drugs that target the process of senescence have become a new therapeutic strategy for the early prevention and latter treatment for degenerative diseases. Evidence suggest that whether through genetically modified strategy (Baker et al., 2011; Liu et al., 2019) or chemotherapy (Zhu et al., 2015; Amor et al., 2020), the elimination of p16^INK4a^ senescent cells has been shown to significantly extend the healthspan in murine. So, optimizing treatments to reduce senescence or eliminate senescent cells may exert positive effects on human health.

Senolytic represents a wide range of drugs or small molecules that can selectively eliminate senescent cells. Zhu et al. (2015) demonstrated a significant effect of senolytic on aging-related diseases in mice for the first time in 2015. Since then, senolytic has been investigated in age-related diseases extensively. Its characteristic of selectively inducing apoptosis of senescent cells has been validated in osteoporosis, primary biliary cholangitis, atherosclerosis, and chronic lung diseases, respectively (Childs et al., 2016; Farr et al., 2017; Barnes et al., 2019; Sasaki et al., 2020). Therefore, the application of senolytic drugs is a potential strategy for degenerative disease treatment, including IVDD.

## Cellular Senescence

Cellular senescence is usually defined as the process of gradual decline in cell proliferation and differentiation as well as physiological dysfunctions during life activities of cells (van Deursen, 2014). The dynamic balance of cell senescence, death, and cell proliferation is fundamental for normal metabolic activities (Calcinotto et al., 2019). Currently, it is widely believed that cellular senescence can be divided into two categories, replication senescence (RS) and stress-induced senescence (SIPS) (Muñoz-Espín and Serrano, 2014). The signaling pathways involved in these two types of senescence overlap with each other a lot, which makes it difficult to distinguish unless external predisposing factors are clarified (Herranz and Gil, 2018).

The mechanism of age-related replication senescence has been studied for decades, and a number of hypotheses have been proposed (Victorelli and Passos, 2017; Schmeer et al., 2019). Among them, the theory of aging circadian clock generally accepted by scientists claims that shortened chromosomal telomeres caused by DNA duplication during mitosis lead to senescence (Dudek et al., 2017; Hood and Amir, 2017; Sato et al., 2017). The reactive oxygen species (ROS) theory describes that the accumulation of ROS in cells will cause organelle dysfunction (Finkel and Holbrook, 2000; Victorelli and Passos, 2017; Wang R. et al., 2018). And, the theory of DNA damage accumulation is also used to explain the mechanism of RS (Lieber and Karanjawala, 2004; Chen et al., 2007). As for SIPS, which refers to cell senescence induced by external stimuli, it manifests as the senescence of local tissues or cells instead of the whole organism. Accordingly, SIPS is considered to be the main cause of age-related degenerative diseases (de Magalhães and Passos, 2018).

Non-random damage plays an important role in the initiation and development of senescence (Gladyshev, 2013). ROS, DNA lesions, and mitochondrial dysfunction caused by external stimulation have been shown to be the dominant mechanisms of SIPS (Passos et al., 2007). As one of the common causes leading to SIPS, DNA damage induced by external stressors mainly activates DNA damage response (DDR) signaling pathway-related kinase (ATM, ATR), which further activates the downstream p53-p21-retinoblastoma protein (Rb) pathway and thereby inhibits cell cycle (Gladyshev, 2013; Ou and Schumacher, 2018). Extensive mechanical tension represents a common external stressor, and the senescence of NP and AF cells causes a typical feature in degenerative intervertebral disc (Pang et al., 2017; Zhao et al., 2019). Additionally, due to the absence of blood vessels and nerves in the intervertebral disc, it is more susceptible to be damaged by oxidative stress, lower pH, hypertonic extracellular environment, and hypoglycemia (Huang et al., 2014; Che et al., 2020). Oxidative stress and hypertonic extracellular environment have been shown to promote NP cell senescence *in vitro* (Xu et al., 2019; Che et al., 2020).

When external stressors cause DNA damage, the DDR signaling pathway and p16^Ink4a^ are both continuously activated (Rodier et al., 2009; Wang et al., 2013; Horikawa, 2020). Moreover, mitochondrial dysfunction and ROS production are also regulated through p38-MAPK pathway. They can then lead to cell cycle arrest, also known as cell senescence (Passos et al., 2007; Borodkina et al., 2014). Recent studies have shown that the inhibition of DDR-related kinase could get senescent cells back on the track of replication cycle instead of cycle arrest (Menolfi and Zha, 2020; Zhao et al., 2020). The significance of DDR is to restrict the damaged DNA to stimulated cell by inhibiting cell proliferation, which attenuates the accumulation of abnormal daughter cells with damaged DNA in the tissue (Lans et al., 2019).

Oxidative stress (OS) is a negative process with the production of ROS, which is a key factor leading to senescence and extensive cellular dysfunction (Junqueira et al., 2004; Hernandez et al., 2015). Hypoxia is the main cause of ROS production (Giordano, 2005). Increasing ROS in cells is closely related to DNA damage, and DDR-related p53-p21-Rb pathway is subsequently activated (Passos et al., 2007). The role of the p16-Rb pathway has also been validated in ROS-induced senescence (Rayesse et al., 2012).

Another important factor of SIPS is mitochondrial dysfunction-induced senescence, whose underlying mechanism is closely related to ROS (Bhatti et al., 2017). On the one hand, mitochondria, as the center of aerobic respiration, are manufacturers of ROS through the loss of the respiratory chain, the decrease of mitochondrial membrane potential, and electronic leak due to opening of mitochondrial permeability transition pores (Sas et al., 2018). On the other hand, mitochondria are the main targets of ROS in turn (Bhatti et al., 2017). When mitochondria are attacked by ROS, mutations of its DNA (mtDNA) increase and respiratory enzymes are damaged, subsequently leading to severe mitochondrial dysfunction (Bhatti et al., 2017; Stokman et al., 2017). Consequently, ROS and mitochondrial dysfunction form a vicious cycle of mutual deterioration (Passos et al., 2007). Furthermore, Wiley et al. (2016) have found that mitochondrial dysfunction-related senescence activated by AMPK and p53 axis produces a completely different SASP compared to that of the IL-1-dependent group, providing new evidence to explain the senescence induced by mitochondrial damage. This result elucidates that mitochondrial dysfunction has multiple effects on cell senescence, while the underlying mechanism needs to be further clarified. Besides hypoxia induced by DNA damage, ROS can also be generated by oxidative stress connected to inflammation and high glucose (McGarry et al., 2018; Fouda et al., 2020).

From the studies above, we conclude that the p53-p21-Rb pathway or/and the p16^INK4a^-Rb pathway play a key role in SIPS. Although these two pathways prevent cells from entering the S phase by inhibiting the phosphorylation of Rb through different cyclin-dependent kinases (CDKs), the cells finally manifest a similar senescence phenotype (Panossian et al., 2021). The difference between two pathways is that p21 is an inhibitor of CDK2 (Morisaki et al., 1999), while p16^INK4a^ works as the inhibitor of CDK 4/6 (Tao et al., 2017). Moreover, the tumor suppressor p19^Arf^, which could activate p53, plays a role in replicating DNA damage instead of acute damage (Lim et al., 2021). Although p53 is essential to the initiation of senescence (Horikawa, 2020), studies have found that p16^INK4a^ is the main factor maintaining senescence in some cells (Baker et al., 2011). Studies on intervertebral disc have only found increased expression of p16^INK4a^ with increasing age, while the levels of p53 and p19Arf did not manifest a significant discrepancy, which emphasized the importance of p16^INK4a^ as a key marker for maintaining age-dependent senescence in the intervertebral disc of mice (Novais et al., 2019).

In terms of transcriptome, miR-623 has been shown to prevent LPS-induced senescence of intervertebral disc cells through CXCL12 (Zhong et al., 2021).

Currently, SIPS is widely recognized as a typical stress response when suffering from external stimuli, which is involved in the repair of damaged tissue and inhibition of abnormal proliferation (Sikora et al., 2011; Dominic et al., 2020). With risk factors affecting the organization continuously, senescent cells will accumulate and bring about unexpected dysfunctions subsequently (de Magalhães and Passos, 2018). Studies have also found that senescent cells play an irreplaceable role in degenerative diseases such as IVDD through triggering extensive inflammatory responses by secreting SASP (Ngo et al., 2017).

## Senescent Cells Play an Indispensable Role in Intervertebral Disc Degeneration

**FIGURE 1:**
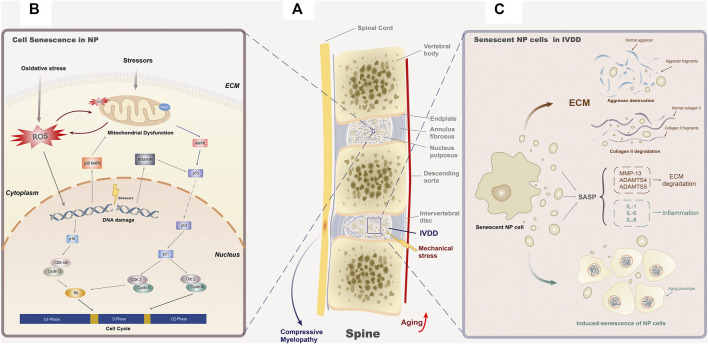
Cellular senescence and IVDD. **(A)** Age-related senescence often leads to organ dysfunction and structure failure, while in the spine, it mainly manifested as IVDD. The senescence of NP cells and fibroblasts in the annulus fibrosus cells is the main cause of IVDD. Under the exposure of inappropriate mechanical stress, the degenerated intervertebral disc may lose its stability as a consequence of the annulus fibrosus enlargement or even rupture and the position changes of the nucleus pulposus, resulting in posterior disc protrusion and subsequent compressive myelopathy. **(B)** Senescence of NP cells is considered to be the main cause of IVDD. Various external and internal stressors can activate the aging-related signal cascade by changing the function of mitochondria, inducing ROS generation or DNA damage. These three initiation modes of senescence are interrelated to each other, forming a complex cellular senescence regulation network. In the downstream cascade, p16-CDK4/6-Rb and p53-p21-CDK2-Rb are recognized to block the G1/S phase of the cell cycle, while p53-p21-cdc2 blocks the S/G2 phase. **(C)** SASP secreted by senescent NP cells will result in structural degradation of the intervertebral disc. SASP in nucleus pulposus is mainly composed by two categories of elements, inflammatory factors (IL-1, IL-6, and IL-8) and ECM proteases (MMP-13, ADAMTS4, and ADAMTS5). For inflammatory factors, they participate in the local inflammatory response and induce the senescence phenotype of adjacent cells. As for ECM proteases, they mainly degrade the components of the extracellular matrix, aggrecan (ADAMT4 and ADAMT5), and collagen II (MMP13), to threaten the homeostasis of extracellular structure.

As we have mentioned in the *Introduction*, aging is the most important factor that promotes the initiation and development of IVDD. Besides apoptosis, NP cells affect adjacent tissues in an aging-related way and aggravate the dysfunction of the intervertebral disc with our body becoming old generally (Wang et al., 2016). Among them, senescent cells are involved in the etiology of several age-related dysfunction, including Alzheimer’s disease (Lyons and Bartolomucci, 2020), obstructive pulmonary disease (Barnes et al., 2019), atherosclerosis (Stojanović et al., 2020), and osteoporosis (Khosla, 2013). Studies have found that the percentage of senescent cells experienced a significant increase in the nucleus pulposus during the process of IVDD (Zhang Y. et al., 2020). Senescent cells exhibit three main characteristics *in vivo*, including reduced replication, expression of SASP, and p16^INK4a^ activation (Li et al., 2021). As the reduction of senescent cell replication has been elaborated in detail in the *Cellular Senescence* section, we will review the two remaining features in the following section (Figure 1).

### Senescence-Associated Secretory Phenotype and Intervertebral Disc Degeneration

ECM degradation and the initiation of inflammation are the two major processes of IVDD (Yang S. et al., 2020). ECM, mostly composed of aggrecan and collagen type II (collagen II), provides a foster ground for the metabolism of intervertebral disc cells (Zhang Y. et al., 2020). However, with the body getting older, the synthesis of aggrecan and collagen II decreases year by year. These degenerative changes could alter the microenvironment of the NP cells, leading to changes of intervertebral disc structure and subsequently weakened load capacity (Smith et al., 2011). In addition, researchers have found that the ability of senescent NP cell to stabilize ECM declines with age (Vo et al., 2016). When subjected to the stimulation of external ROS, aggrecan and collagen Ⅱ are degraded as a consequence of increased expression of matrix metalloproteinase family (MMPs) and A Disintegrin And Metalloproteinase with Thrombo Spondin 1 repeats (ADAMTSs), which is considered to be the main microscopic pathological change of IVDD. (Bedore et al., 2014).

Inflammatory factors (IL-1, IL-6, IL-8, and TNF-α) are key cytokines involved in aging and age-related diseases, whose expression levels are related to the severity of degeneration to a certain extent (Krupkova et al., 2018; Zhang Y. et al., 2019; Kim et al., 2021). IL-1 has already been shown to be involved in the pathogenesis of intervertebral disc degeneration (Phillips et al., 2013). But, the research of Gorth et al. (2019) suggests that there is also an exception in IVDD. After knocking out gene IL-1 in mice, they found IVDD was remarkably exacerbated compared to the control group, which suggests the opposite effect of IL-1 in intervertebral disc. Therefore, it is worth noting that the function of inflammatory factors clarified in other degenerative diseases cannot be simply applied to IVDD. In the past few years, it is widely believed that senescent cells can play multiple roles, including but not limited to secreting metabolic factors, recruiting inflammatory cells, causing instability of extracellular microenvironment, and inducing the senescence of neighboring cells through paracrine. In this way, senescent cells could lead to the catabolism of the intervertebral disc matrix and activated inflammation, which are two main mechanisms of IVDD (McHugh, 2020; Yang S. et al., 2020).

The metabolic factors secreted by senescent cells are called SASP, which can be divided into proinflammatory factors (IL-6, IL-7, IL-8, and TNF-α), ECM proteases (MMPs and ADAMTS), growth factors, cytokines, and other biologically active substances (Faget et al., 2019; Basisty et al., 2020). ATM/ATR activated by DDR is considered to be an important target for initiating SASP synthesis (Victorelli and Passos, 2017; Menolfi and Zha, 2020), among which the silencing of ATM genes can remarkably inhibit the secretion of IL-6 (Rodier et al., 2009). The theory has also been validated in IVDD, and inhibition of ATM alleviated the symptom significantly with reducing the expression of IL-6 in SASP (Zhao et al., 2020). In addition, tumor suppressor genes have been confirmed to play a role in the initiation of cellular senescence, especially for the secretion of SASP. In astrocytes, p53 regulated the expression of a variety of SASP factors (IL-1β, IL-6, and IL-8) (Turnquist et al., 2016), while another tumor suppressor gene Rb promoted the activation of the SASP (IL-6) in the mouse osteosarcoma model (Li and Zhang, 2017). Moreover, mammalian rapamycin (mTOR) (Herranz et al., 2015), p38MAPK/MAPK-activated protein kinase 2 (MK2) (Freund et al., 2011), nuclear factor kappa-B (NF-κB) (Chien et al., 2011; Zhao et al., 2020), interferon gene cyclic GMP-AMP synthetase/stimulator (cGAS/STING), and CCAAT/enhancer binding protein β (C/EBPβ) have been confirmed to regulate the secretion of SASP (Huggins et al., 2013; Lim et al., 2020; Aguado et al., 2021).

The secretion of SASP causes a variety of extracellular reactions, in which inflammation is the most important one. Accordingly, the interaction between the immune system and cellular senescence provides a new direction for the diagnosis and treatment of age-related diseases. A contradiction is that SASP could specifically eliminate senescent tumor cells by recruiting immune cells (NK cells) (Chien et al., 2011), meanwhile SASP could also cause the “senescence cascade” as more cell senescence is induced by endocytosis of pro-inflammatory cytokines (Faget et al., 2019).

In IVDD, how SASP plays its role has attracted widespread attention currently. It has been determined that the expression level of multiple pro-inflammatory cytokines (IL-1, IL-6, and IL-8) and ECM proteases (MMP-13, ADAMTS4, and ADAMTS5) increased in senescent intervertebral disc (Ngo et al., 2017). When aging-related degeneration occurs, ECM is degraded by elevated ECM proteases (MMPs and ADAMTS), which is an important mechanism for the loss of structural stability of the intervertebral disc (Bedore et al., 2014).

At present, some drugs called senomorphics are found to improve the extracellular microenvironment by reducing the production of SASP without eliminating senescent cells (distinguish from senolytics) (Di Micco et al., 2021). In some literature, they are also called senostatics (Kang, 2019). Our previous result suggested that the senomorphic drug metformin and polydatin could alleviate IVDD by inhibiting the senescence and apoptosis of NP cells in intervertebral disc (Chen et al., 2016; Wang J. et al., 2018), and the inhibitory effect of metformin on SASP (IL-6, IL-1β, MMP3, and MMP13) was recently verified in the study of Han et al. (2019).

However, the composition of SASP varies from cell to cell (Basisty et al., 2020), and even for the same cell, the component of SASP it produces is partially different depending on the type of stimuli it receives and for which it enters senescence (Özcan et al., 2016). On this condition, components of SASP secreted by senescent NP cells are still not fully understood. On the other hand, as we mentioned before, SASP can recruit immune cells for self-elimination (Birch and Gil, 2020; Jin et al., 2021), while it is still unclear whether the mechanism exists in the intervertebral disc. So, further implementation of senomorphic drugs in IVDD is limited, although the deteriorating effect of SASP in IVDD has already been clarified (Zhao et al., 2011; Ngo et al., 2017). Analogically, the application of senolytic drugs that aim to remove senescent cells and reduce SASP secretion in intervertebral disc requires further research.

### Eliminating Senescent Cells and Relieving Intervertebral Disc Degeneration

Since the accumulation of senescent cells is the main cause of age-related degenerative diseases, it is widely believed that removing senescent cells and reducing the production of SASP can not only alleviate the aging-related dysfunction but also improve age-related diseases or related phenotypes (Baker et al., 2011; Zhu et al., 2015). For example, removing senescent astrocytes and microglia has been shown to improve tau-dependent neurodegenerative diseases (Zhang P. et al., 2019), while eliminating senescent osteocytes can also delay osteoporosis caused by aging effectively (Farr et al., 2017). Similar effects have also been observed in chronic lung diseases (Barnes et al., 2019) and atherosclerosis (Childs et al., 2016) after senescent cells being eliminated. In the phase of pre-clinical research, selective elimination of transgenic marker p16^INK4a^ and chemical pathways are two effective strategies to remove senescent cells (Baker et al., 2011; van Deursen, 2019).

p16^INK4a^, known as a cell cycle suppressor, can inhibit cyclin-dependent kinases 4 and 6 (CDK4 and CDK6), causing stagnation of senescent cells at G1 phase (Kim et al., 2021). The expression level of p16^INK4a^ increases in senescent cells; thus, p16^INK4a^ is always used as one of the commonly senescent cell markers (others: SA-β-gal, ki67) (Krupkova et al., 2018; Ogrodnik, 2021). In recent years, more and more studies have proved that p16^INK4a^ is essential for inducing and maintaining senescence in intervertebral discs, and its conditional loss plays an important role in reducing cell apoptosis, restricting SASP, and changing matrix homeostasis of intervertebral disc cells (Liu et al., 2019; Novais et al., 2019; Patil et al., 2019; Che et al., 2020). After inserting a strain (tdTOM) into the p16 promoter, Liu et al. (2019) have found that p16^tdTOM^-positive cells accumulate accompanying aging and inflammation, among which mouse macrophages exhibited a senescent phenotype, and SASP-related expression was significantly elevated. Also, Che et al. (2020) detected the decrease of pro-inflammatory cytokine IL-1β and IL-6 after knocking out p16^INK4a^. Although a decreased expression in MMP13 was not detected, the inhibited degradation of collagen II in the intervertebral disc indicated that knocking out p16^INK4a^ can prevent intervertebral disc from inflammatory response and matrix degradation through SASP. Interestingly, in the study by Novais et al. (2019), although the secretion of SASP was reduced and ECM homeostasis was improved in p16^INK4a^ KO mice, there was no significant difference in the reduction of senescent cells in the intervertebral disc and the improvement of IVDD process compared with the control group. Instead, the increase of p19Arf and Rb expression suggests that perhaps some bypasses are activated when p16^INK4a^ is silenced, maintaining the senescent phenotype. This result suggests that inhibiting the expression of p16^INK4a^ merely cannot alleviate IVDD effectively.

Alternatively, the elimination of senescent cells has been proved to decrease the paracrine effect of SASP on neighboring cells (Ngo et al., 2017), which exhibits few obvious side effects simultaneously (Baker et al., 2011). Furthermore, after the elimination of transgenic p16^INK4a^ (p16-3MR) senescent NP cells by target reagents, Patil et al. (2019) have detected the extensive reduction of MMP13 expression and ECM degradation in the intervertebral disc. Although the expression of IL-6 and ADAMTS4 exerts no distinction compared with negative control, which suggests that IL-6 and ADAMTS4 may not be the SASP components that cause the development of IVDD, the targeted elimination of senescent cells significantly improves the histological characteristics of the intervertebral disc compared with the knockout of p16^INK4a^. The results suggest that the elimination of senescent NP cells with a high expression of p16^INK4a^ can delay the aging process and reduce the degeneration of intervertebral discs.

A senolytic is a kind of small molecules with the feature of clearing senescent cells selectively (van Deursen, 2019). After dasatinib and quercetin were first reported as a senolytic combination in 2015, it was discovered that the indiscriminate removal of senescent cells through senolytic can effectively inhibit the degradation of proteoglycans in the intervertebral disc (Zhu et al., 2015). Dozens of compounds with senolytic activity have been distinguished so far, which are mainly divided into natural extracts with their derivatives and synthetic chemical molecules (Partridge et al., 2020; Lagoumtzi and Chondrogianni, 2021).

As for natural extracts, compounds with senolytic features mainly include quercetin, fisetin, curcumin, and its derivative o-Vanillin (Di Micco et al., 2021). The latest study has found that the grape seed extract PCC1 also has senolytic properties, which may be stronger than existing compounds (Xu Q. et al., 2021). Among them, curcumin and its metabolite o-Vanillin have been proven to eliminate senescent cells in the intervertebral disc, reducing SASP secretion and alleviating IVDD (Cherif et al., 2019; Mannarino et al., 2021). Our previous study has elucidated that quercetin could significantly reduce the proportion of senescent cells in the intervertebral disc through the Nrf-2/NF-κB pathway and slow down the deterioration of IVDD (Shao et al., 2021). The antiaging effects of fisetin and PCC1 in the intervertebral disc reserve to be proven.

For synthetic chemical molecules, the effects of FDA-approved reagent RG7112 in eliminating senescent cells were validated in the intervertebral disc by targeting MDM2 (Cherif et al., 2020). From the preclinical studies above, we have seen the feasibility and great potential of senolytic therapy in IVDD. Compared with transgenic therapies, senolytic drugs have fewer side effects and clearer mechanisms. Also, some of them are clinical drugs that have been approved by the FDA, which have better prospects in application (Nogueira-Recalde et al., 2019; Sargiacomo et al., 2020). However, considering the beneficial effects of senescent cells in some diseases such as tissue repairing (Xu et al., 2018; Lagoumtzi and Chondrogianni, 2021), the systemic application of senolytic drugs needs a more in-depth evaluation.

## Potential Intracellular Targets of Senolytic and Intervertebral Disc Degeneration

**FIGURE 2:**
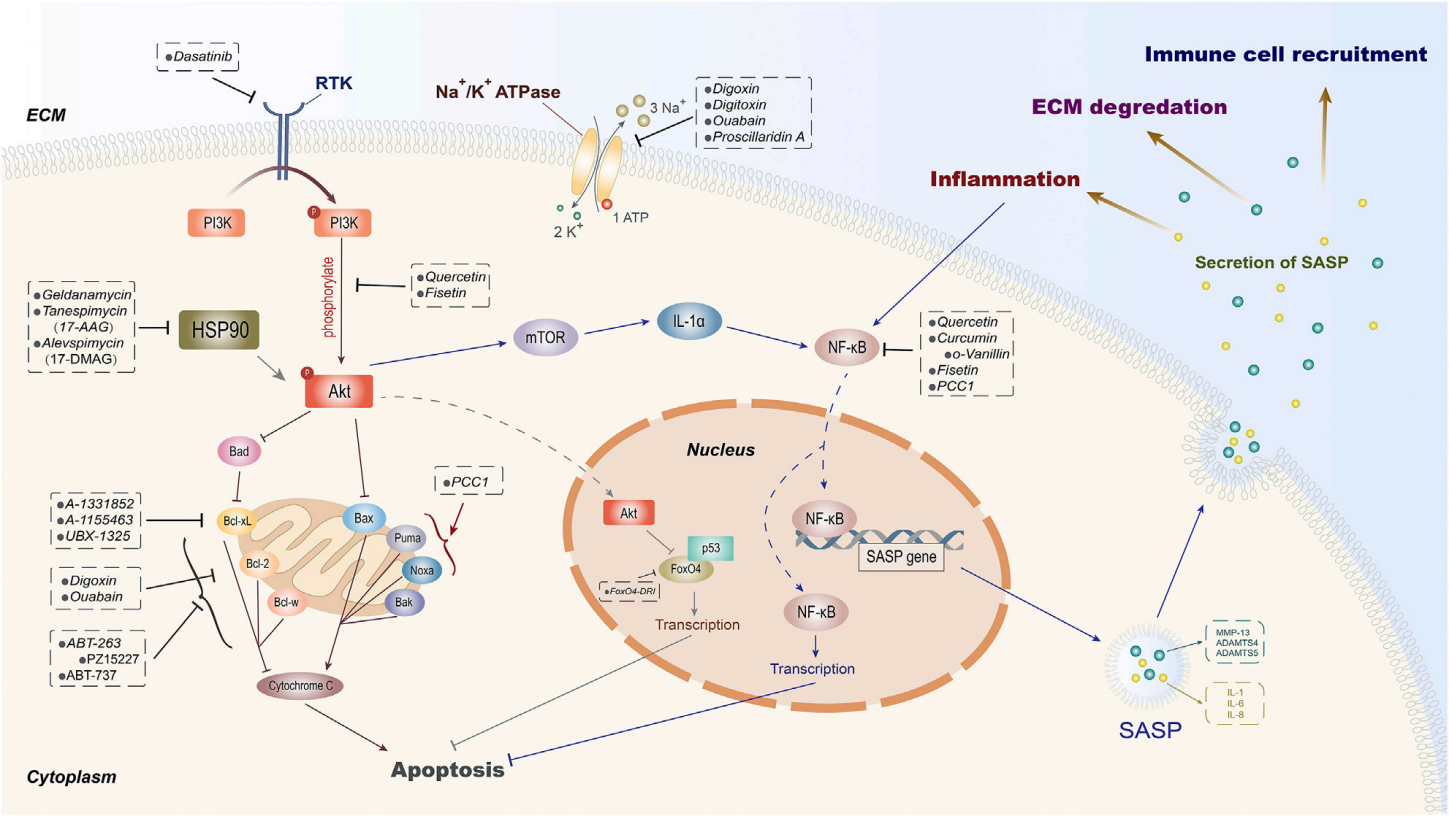
Potential intracellular targets of senolytics. According to the diverse intracellular targets of different senolytics, they are divided into certain groups. As one of the principal pathways targeted by senolytics, PI3K/Akt can be suppressed by dasatinib through receptor tyrosine kinase (RTK). The natural extracts quercetin and fisetin are proven to inhibit the phosphorylation of Akt and then induce senescent cell apoptosis and SASP-secret inhibition through FoxO4 and mTOR, respectively. Besides quercetin, curcumin with its derivative o-Vanillin and fisetin exert their senolytic effects by inhibiting NF-κB. HSP90 inhibitors have also been shown to induce apoptosis by indirectly inhibiting Akt. The FDA-approved cardiac glycoside drugs digoxin, digitoxin, and ouabain can mediate the elimination of senescent cells by inhibiting Na+/K+ ATPase and anti-apoptotic protein Bcl-2 simultaneously. In addition, senolytic properties of inhibitors on anti-apoptotic proteins (Bcl-2, Bcl-w, and Bcl-xL) of Bcl-2 family and transcription factor FoxO4 have also been demonstrated.

In recent years, an increasing number of studies have shown that the application of transgenes or chemical reagents in eliminating senescent cells can protect organisms from aging and some age-related degenerative diseases and prolong the healthspan (Baker et al., 2011; van Deursen, 2019). As a type of aging treatment strategy, senolytics can selectively clear senescent cells by inducing apoptosis and reduce the production of SASP, contributing to the alleviation of aging-related diseases and improvement of metabolic function (Xu et al., 2018; Lagoumtzi and Chondrogianni, 2021). The naturally extracted senolytic has a complicated mechanism where multiple intracellular targets are involved (Ge et al., 2021). For synthetic senolytics, there are four types of potential targets that have been identified in senescent cells, including BCL family (Yosef et al., 2016), HSP90 inhibitor (Fuhrmann-Stroissnigg et al., 2017), PI3K/AKT (Wagner and Gil, 2020), and others (Hubackova et al., 2019; He et al., 2020a; Zhang et al., 2021). Here, we will discuss the underlying mechanisms of these senolytic target families on cell senescence as well as their role in the process of IVDD (Figure 2).

### PI3K/Akt

PI3K (phosphatidylinositol kinase) is a dimer that is composed of regulatory subunit p85 and catalytic subunit p110 (Cully et al., 2006). When binding to growth factors, cytokines, or hormone receptors, PI3K can change the structure of Akt and make it phosphorylated (p-AKT). p-AKT is the activated state of Akt that could regulate FoxO, mTOR, and other signal proteins in its downstream (Yang et al., 2019). PI3K/Akt is a classic pathway that plays an important regulatory role in cell proliferation, apoptosis, and autophagy (Koyasu, 2003; Cully et al., 2006; Greenhill, 2019; Zhou et al., 2020). Meanwhile, an increasing number of evidence show that the activation of the PI3K/AKT pathway is also involved in stabilizing senescent cells (Zhu et al., 2015; Mendez-Pertuz et al., 2017; Wagner and Gil, 2020).

Until now, two types of downstream targets have been figured out in inhibiting the apoptosis of senescent cells through PI3K/Akt.

The first is the pro-apoptotic signaling pathways mainly suppressing Bax, Bad, and FoxO (Stitt et al., 2004; She et al., 2005; Rahmani et al., 2018). Studies have found that after blocking the binding of FoxO4 and p53, FoxO4-DRI can induce the apoptosis of aging Leydig cells in testicles effectively (Zhang C. et al., 2020), while the role of Bax and Bad inhibitors in inducing apoptosis of senescent cells has not been reported yet.

mTOR, together with NF-κB signaling pathways, represents another kind of targets regulated by PI3K/Akt (Dutta Gupta and Pan, 2020). As an important regulatory protein in aging, autophagy, and apoptosis, mTOR plays a key role in cell senescence and autophagy (Nam et al., 2013; Kim and Guan, 2015). mTORC1 is widely involved in cell metabolisms whose suppression will lead to the surging autophagy and clearance of damaged proteins and organelles (such as mitochondria) (Weichhart, 2018), which could also reduce the accumulation of SASP and alleviate aging (Weichhart, 2018). The application of rapamycin, inhibitor of mTORC1, has been shown to extend the lifespan of model organisms, although the latest clinical trial results do not support its anti-aging effect in healthy older adults (Kraig et al., 2018). Accordingly, whether mTORC1 inhibitors can alleviate human aging needs further discussion. As in intervertebral disc, rapamycin can exert a variety of effects through mTORC1, including inhibiting the senescence of annulus fibrosus cell (Gao et al., 2018), promoting autophagy of end plate chondrocytes (Zhang C. et al., 2020), and protecting NP cell from apoptosis and senescence (Kang et al., 2019). Hence, the process of IVDD was delayed (Kakiuchi et al., 2019). Due to its properties in reducing SASP secretion, rapamycin is classified as senomorphic currently (Xu C. et al., 2021).

NF-κB is another important molecule regulated by PI3K/Akt (Hoesel and Schmid, 2013). We have validated the role of NF-κB in reducing the production of SASP and elimination of senescent NP cell under the treatment of quercetin (Shao et al., 2021). Furthermore, quercetin has also been shown to inhibit the upstream of PI3K/Akt pathway through certain mechanisms, suppressing the proliferation of tumor cells and inducing the apoptosis of senescent cells (Kim et al., 2019; Lan et al., 2019). In addition, another natural senolytic drug fisetin is also proven to promote apoptosis of tumor and senescent cell by inhibiting PI3K/Akt and mTOR (Yousefzadeh et al., 2018). Therefore, PI3K/Akt is considered to be an essential target of natural senolytic extracts as well as the potential signaling pathway for IVDD.

### B-Cell Lymphoma 2 Family

The B-cell lymphoma 2 (BCL-2) family is a special protein family that shares one or more BCL-2 homology (BH) domains. Most of BCL-2 are located on the outer mitochondrial membrane or transferred to outside of the mitochondria under certain stimuli (Adams and Cory, 1998). The family plays a decisive role in the intracellular apoptosis by regulating the permeability of the outer mitochondrial membrane (Korsmeyer, 1999).

At present, 25 proteins have already been recognized as Bcl-2 family homologue (Kale et al., 2018). According to different responses to apoptotic stimulus, they can be divided into two groups: Bcl-2, Bcl-xL, Bcl-w, MCL-1, and A1 who inhibit cell apoptosis and Bax, Bak, Bad, Noxa, etc. that promote cell apoptosis (Adams and Cory, 1998). Bcl-2 can prevent the release of cytochrome c from mitochondria to the cytoplasm, thereby inhibiting endogenous cell apoptosis (Chipuk et al., 2004; Sun et al., 2019). Besides, Bcl-2 also plays an important role in the survival of senescent cells (Chong et al., 2020). Studies have found that the expression of anti-apoptotic proteins Bcl-2, Bcl-xL, and Bcl-w increased, which enhances the cells’ ability of drug resistance through cell senescence by anti-apoptotic pathway and hence resisting external stimuli and maintaining tissue integrity (Yosef et al., 2016). This evidence indicates that anti-apoptotic proteins in the BCL-2 family have become potential targets for senolytic drugs.

Since inhibiting the expression of anti-apoptotic proteins such as BCL-2, BCL-xL, and BCL-w displays a positive effect on tumor cell apoptosis, a series of anti-cancer reagents targeting BCL-2 have been developed in recent years (Delbridge et al., 2016). For senescence, the senolytic properties of some BCL-2-targeting molecules [such as ABT263 (navitoclax), ABT737, and A-1331852] have been verified in a few systems (Chong et al., 2020; Sasaki et al., 2020; Yang H. et al., 2020). Another BCL-XL inhibitor A-1155463 has not been applied in the study of senescent cell clearance yet (Xu D. et al., 2021).

Interestingly, previous studies in IVDD have found that the apoptosis of normal functioning cells in the nucleus pulposus and endplate is an essential element to degeneration’s exacerbation (Chen et al., 2018; Xie et al., 2019), and researchers used to activate Bcl-2 by miRNA or chemical activators to inhibit the apoptosis and alleviate IVDD caused by apoptosis (Chen et al., 2015; Song et al., 2018). Given the contradiction, the challenge turns into how to guide Bcl-2 inhibitors to induce apoptosis in senescent cells exclusively instead of normal cells, which is the key to reducing the side effects of Bcl-2 inhibitors.

The latest study by Lim et al. (2021) elucidates that the BCl-2 inhibitor ABT-263 (navitoclax) did not display any interference to normal functioning cells while inducing the apoptosis of senescent NP cells in intervertebral disc. However, this study did not elucidate whether apoptosis of normal functioning cells occurred in the process of ABT-263 administration and whether the positive effect was dose related. In order to prevent ABT-263 (navitoclax) from acting on other tissues of the body, researchers loaded ABT-263 (navitoclax) in poly(lactic-co-glycolic acid) nanoparticles (PLGA-ABT), which can specifically release the reagent to the intervertebral disc. After the release of ABT-263, the secretion of SASP (IL-6 and MMP-13) in the intervertebral disc was significantly reduced, the degradation of ECM was weakened, and the structural integrity and stability of the intervertebral disc were restored as well (Lim et al., 2021). Besides ABT-263, several studies have also proven that some senolytic drugs could induce apoptosis of senescent disc cells *in vitro* and *in vivo*, providing inspirations for the application of BCL-2 inhibitor drugs in the treatment of IVDD (Wang et al., 2016; Xu et al., 2018; Wu et al., 2020). Although the study of Lim et al. has enlightened the application of senolytic in IVDD to a certain degree, how to accurately guide BCL-2 inhibitors to senescent cells in the intervertebral disc and adjust the dosage so that the positive effects of BCL-2 inhibitors by inducing the apoptosis of senescent cells far outweigh the negative effects caused by normal functioning cell apoptosis requires further research.

### HSP90

Heat shock protein (HSP) refers to a protein group produced under the stimulation by stressors, especially for high-temperature environment (Richter et al., 2010). The majority of HSP family are known as molecular chaperones, which can assist proteins to fold correctly. In light of molecular weight, HSPs are divided into five categories: HSP110, HSP90, HSP70, HSP60, and small heat shock proteins (sHSPs) (Saibil, 2013). HSP90 plays a vital role in stabilizing key signal proteins from heat stress whose inhibition will lead to apoptosis in tumor cells (Pungsrinont et al., 2020; Khaledian et al., 2021). As a consequence, HSP90 has been intensively studied as a target of cancer chemotherapy, and some anti-tumor compounds that inhibit HSP90 have been developed.

In recent years, the role of HSP90 in cell senescence has aroused a wide interest. Currently, in nasopharyngeal carcinoma, age-related cardiac disorders, age-related macular degeneration, and Alzheimer’s disease, the increase of HSP90 expression in senescent cells has been verified, and the senolytic activity of HSP90 inhibitors has also been recognized (Chan et al., 2013; Carnemolla et al., 2014; Wang et al., 2014; Fuhrmann-Stroissnigg et al., 2017; Chen et al., 2021; Criado-Marrero et al., 2021). Studies have found that HSP90 can activate Akt through phosphorylation and inhibit the apoptosis of senescent cells (Chen et al., 2017). In addition, according to what we have introduced before, DNA damage caused by radiation or external and internal factors is an important part of cell senescence and even apoptosis (Dote et al., 2006), and the expression of HSP90 increases during this process, which stabilizes the DDR reactive proteins in senescent cells (Solier et al., 2012; Orth et al., 2021).

Currently, the senolytic activity of HSP90 inhibitors has been gradually proven. For example, HSP90 inhibitor ganetespib has shown obvious senolytic effects in the treatment of prostate cancer (Pungsrinont et al., 2020). Besides anti-cancer effects, further studies on HSP90 inhibitors are suggested to evaluate its senolytic potential (Fuhrmann-Stroissnigg et al., 2017). In IVDD, inhibiting HSP90 in nucleoplasmic stem cells/progenitor cells (NPSCs) has been shown to alleviate the process, but its effect on the clearance of senescent cells in the intervertebral disc needs further exploration (Hu et al., 2020).

### Others

SirT1 is a deacetylase that can promote autophagy and suppress cell apoptosis and inflammation by inhibiting the p53-p21-Rb axis (Rubinsztein et al., 2011). SirT1-autophagy pathway’s activation can effectively slow down the senescence of AF and NP cells in the intervertebral disc (Zhou et al., 2016; Zhu et al., 2021). Except for NF-κB, SirT1 pathway has also been proven to be one of quercetin’s aging-resistant targets (Lazo-Gomez and Tapia, 2017; Liu et al., 2020). The study of Wang D. et al. (2020) has proven that quercetin can inhibit apoptosis and relieve IVDD by activating SirT1 in the intervertebral disc *in vivo* and *in vitro*. Resveratrol, a strong activator of SirT1, is also proven to alleviate IVDD (Li et al., 2019). However, its anti-apoptotic properties suggest that it should be classified as senomorphic senomorphic/senostatic, revealing that SirT1 may be a senormorphic’s target instead of senolytic’s.

In addition, some studies have also found that MDM2, HDAC, OXR1, and USP7 have the potential as senolytic targets (Samaraweera et al., 2017; Zhang et al., 2018; He et al., 2020a; Xu et al., 2020). Whether these targets can be inhibited by senolytics and subsequently decelerate the progression of IVDD needs to be further studied. Unambiguously, research on senolytic targets can provide a molecular basis for us to avoid side effects when applying such drugs in the future.

## Concluding Remarks and Prospects of Senolytic in Intervertebral Disc Degeneration Therapy

In this review, we first sort out the mechanism of cellular senescence, including RS and SIPS. SIPS triggered by external stimuli is considered to be one of the main reactions in IVDD, which includes DDR, oxidative stress, and mitochondrial dysfunction.

Secondly, we discussed the role and mechanism of senescent cells in the process of IVDD through SASP. The p16^INK4a^-Rb and the p53-p21-Rb pathways are considered to be two key signaling axes in regulating SIPS. The expression of p16^INK4a^ in NP and AF cells increased in the intervertebral discs of young IVDD patients, with the telomeres shortened, which proved the intrinsic connection between cellular senescence and IVDD (Le Maitre et al., 2007). At present, it is generally believed that the mechanism of cell senescence affecting the intervertebral disc is as follows. Firstly, senescent NP and AF cells secrete SASP to promote ECM degradation and extracellular inflammation. Then, the intervertebral disc degenerates and loses its stability, causing low back pain and cervical and lumbar spondylosis.

Therefore, senotherapy, which eliminates senescent cells through senolytics or down-regulates the expression of related SASP by senomorphics/senostatics, has become a potential treatment for IVDD (Schmitt, 2017). Compared with surgical treatment and pain-relieving glucocorticoid therapy, the advantage of senotherapy is that it can intervene before the onset of IVDD symptoms. Unluckily, since the components of SASP produced in intervertebral discs are not yet clearly clarified, the application of senomorphics in IVDD remains to be further studied. However, senolytics that reduce the secretion of SASP by eliminating senescent cells have a greater chance of becoming a therapeutic drug for IVDD. Thus, we thirdly summarized several targets of senolytics, including PI3K/Akt, BCL-2 family, and HSP90.

Finally, although several senolytics have been proven to alleviate IVDD in murine, it is still unclear whether the clinical application of senolytics has an effect on the intervertebral disc. Hence, we summarized the intracellular targets of reagents with senolytic properties, reviewed their application in IVDD, and determined their clinical research status and whether they are approved by the FDA (Table 1).

**TABLE 1:** Identified senolytic agents, their targets, whether the role has been validated in IVDD, clinical trial’s status, and whether approved by the FDA.

Senolytic	Targets	In IVDD	Clinical trial’s status	FDA-approved	References
**Natural compounds**					
Quercetin	PI3K/Akt, Nrf2, NF-κB, and SirT1	√	Phase 1 and 2 for COPD (NCT03989271), phase 2 for aging (NCT04946383), and phase 2 for Alzheimer’s disease (NCT04785300)	—	Miao et al. (2021), Shao et al. (2021)
Fisetin	PI3K/Akt, Nrf2, and NF-κB	—	Phase 1 and 2 for osteoarthritis (NCT04210986) and phase 2 for frail elderly syndrome (NCT03675724)	—	Yousefzadeh et al. (2018)
Ouabain	Bcl-2, Noxa, and Na^+^/K^+^ ATPase pump	—	—	—	Triana-Martinez et al. (2019), L’Hôte et al. (2021)
Digoxin	Bcl-2 and Na^+^/K^+^ ATPase pump	—	Phase 2 for rheumatoid arthritis (NCT04834557)	√	Triana-Martinez et al. (2019); Barrera-Vazquez et al. (2021)
Digitoxin	Bcl-2 and Na^+^/K^+^ ATPase pump	—	—	√	Triana-Martinez et al. (2019)
Proscillaridin A	Na^+^/K^+^ ATPase pump	—	—	—	Li et al. (2018), Triana-Martinez et al. (2019)
Curcumin o-Vanillin	Nrf2, NF-κB, and autophagy	√	Phase 1 for age-related macular degeneration (NCT04590196) and phase 2 for Alzheimer’s disease (NCT00099710, completed)	—	Ma et al. (2015), Cherif et al., (2019), Kang et al. (2019)
Piperlongumine	OXR-1	—	—	—	Zhang et al. (2018)
PCC1	NF-κB, Noxa, and Puma	—	—	—	Xu Q. et al. (2021)
**Synthetic molecules**					
Dasatinib	PI3K/Akt and RTK (receptor tyrosine kinase)	—	Phase 1 for aging (NCT04994561) and phase 1 and 2 for Alzheimer’s disease (NCT04785300)	√	Salaami et al. (2021)
ABT-263 (navitoclax) PZ15227	Bcl-2, Bcl-xL, and Bcl-w	—	Phase 3 for myelofibrosis (NCT04468984)	—	Gandhi et al. (2011), He et al. (2020b)
ABT-737	Bcl-2, Bcl-xL, and Bcl-w	—	Preclinical studies for ovarian cancer (NCT01440504)	—	Miyao et al. (2020)
A-1331852	Bcl-xL	—	—	—	Sasaki et al. (2020)
A-1155463	Bcl-xL	—	—	—	Wang L. et al. (2020)
UBX-1325	Bcl-xL	—	Phase 1 for neovascular age-related macular degeneration (NCT04537884) and phase 1 and 2 for diabetic macular edema (NCT04857996)	—	Unity Biotechnology (2021)
Geldanamycin	HSP90	—	—	—	Han et al. (2017)
Tanespimycin (17-AAG)	HSP90	—	Phase 1 for multiple myeloma (NCT00113204) and phase 2 for adenocarcinoma of the prostate (NCT00118092)	—	Calero et al. (2017), Lee-Theilen et al. (2021)
Alevspimycin (17-DMAG)	HSP90	—	Phase 2 for breast cancer (NCT00780000)	—	Fuhrmann-Stroissnigg et al. (2017)
Azithromycin	Autophagy	—	Phase 1 for age-related macular degeneration (NCT00831961)	√	Ozsvari et al. (2018)
Roxithromycin	Autophagy	—	Phase 1 for low back pain (NCT00285493)	√	Zhang et al. (2021)
FoxO4-DRI	FoxO4	—	—	—	Alvarez-Garcia et al. (2017)
UBX0101	MDM2 and p32	—	Phase 2 for osteoarthritis (NCT04129944)	—	Jeon et al. (2017)
RG7112	MDM2	√	Phase 1 for hematologic neoplasms (NCT00623870)	—	Cherif et al. (2020)
P5091	USP7	—	—	—	He et al. (2020a)
Panobinostat	HDAC	—	Phase 3 for multiple myeloma (NCT01023308)	√	Samaraweera et al. (2017)
Fenofibrate	PPAR-α	—	Phase 3 for diabetic macular edema (NCT03345901)	√	Nogueira-Recalde et al. (2019)
**Combination**					
Quercetin and dasatinib	PI3K/Akt, Nrf2, and NF-κB	√	Phase 2 for aging (NCT04946383) and Alzheimer’s disease (NCT04685590)	—	Novais et al. (2021)

Since some senolytics are clinically applied drugs, we introduce some early clinical research and application prospects for their senescent cell-eliminating property as follows.

Pre-clinical studies have proven that the application of senolytics to remove senescent cells could lead to tissue renewal and better physical performance. Moreover, several intracellular targets for senolytics have also been discovered, which provide directions for senolytic development and potential clinical applications in aging-related diseases. After discovering the senolytic effects of quercetin and dasatinib, Dr. Kirkland was also the first to put it into clinical trial. They recruited 14 patients with idiopathic pulmonary fibrosis caused by senescent cells and found that the exercise capacity of these patients continues to improve with the in-depth dasatinib + quercetin combination therapy, which is a miracle for patients with pulmonary fibrosis who were considered incurable before (Justice et al., 2019). The subsequent trial of Dr. Kirkland on patients with diabetic nephropathy manifested that senolytics can eliminate senescent cells in the human body as senescent cell markers and the level of SASP decreased significantly (Hickson et al., 2019).

Since senescent cells are also involved in beneficial processes such as wound healing, embryonic development, tissue regeneration, and cancer prevention, long-term use of senolytics may have potential side effects (He and Sharpless, 2017), and the usage plans of senolytics also need further discussion. Should senolytics be used systemically or locally and long-term or short-term? Accordingly, further studies are needed to enhance effectiveness without weakening the positive effects of cellular senescence before it is applied as an anti-aging drug.
